# Clinical Features and Outcomes of Patients with Sarcoidosis-associated Pulmonary Hypertension

**DOI:** 10.1038/s41598-019-40030-w

**Published:** 2019-03-11

**Authors:** Kishan S. Parikh, Talal Dahhan, Leigh Nicholl, Nicole Ruopp, Gina-Maria Pomann, Terry Fortin, Victor F. Tapson, Sudarshan Rajagopal

**Affiliations:** 1 Department of Medicine, Duke University Medical Center, Durham, North Carolina USA; 2 Department of Biostatistics and Bioinformatics, Duke University, Durham, NC USA; 3 Department of Medicine, Cedars-Sinai Medical Center, Los Angeles, California USA

## Abstract

The presence of pulmonary hypertension (PH) significantly worsens outcomes in patients with advanced sarcoidosis, but its optimal management is unknown. We aimed to characterize a large sarcoidosis-associated pulmonary hypertension (SAPH) cohort to better understand patient characteristics, clinical outcomes, and management strategies including treatment with PH therapies. Patients at Duke University Medical Center with biopsy-proven sarcoidosis and SAPH confirmed by right heart catheterization (RHC) were identified from 1990–2010. Subjects were followed for up to 11 years and assessed for differences by treatment strategy for their SAPH, including those who were not treated with PH-specific therapies. Our primary outcomes of interest were change in 6-minute walk distance (6MWD) and change in N-terminal pro-brain natriuretic peptide (NT-proBNP) by after therapy. We included 95 patients (76% women, 86% African American) with SAPH. Overall, 70% of patients had stage IV pulmonary sarcoidosis, and 77% had functional class III/IV symptoms. Median NT-proBNP value was elevated (910 pg/mL), and right ventricular dysfunction was moderate/severe in 55% of patients. Median values for mean pulmonary artery pressure (49 mmHg) and pulmonary vascular resistance (8.5 Woods units) were consistent with severe pulmonary hypertension. The mortality rate over median 3-year follow-up was 32%. Those who experienced a clinical event and those who did not had similar overall echocardiographic findings, hemodynamics, 6MWD and NT-proBNP at baseline, and unadjusted analysis showed that only follow-up NT-proBNP was associated with all-cause hospitalization or mortality. A sign test to evaluate the difference between NT-Pro-BNP before and after PH therapy produced evidence that a significant difference existed between the median pre- and post-NT-Pro-BNP (−387.0 (IQR: −1373.0-109), p = 0.0495). Use of PH-specific therapy may be helpful in selected patients with SAPH and pre-capillary pulmonary vascular disease. Prospective trials are needed to characterize responses to PH-specific therapy in this subset of patients with SAPH.

## Introduction

Sarcoidosis is a systemic disease that affects the parenchyma, interstitium, thoracic lymph nodes, airways and vasculature of the lungs^1^. Pulmonary hypertension is thought to complicate sarcoidosis in 5–28% of patients, and has been reported to be present in up to 74% of patients with advanced sarcoidosis^1–5^. The presence of sarcoidosis-associated pulmonary hypertension (SAPH), classified as World Health Organization group 5 due to its complex/multifactorial mechanisms^6^, is known to worsen outcomes^5,7,8^. In one case series, the diagnosis of SAPH carried a 7-fold increased risk for death over 3-year follow-up^3^.

No pulmonary arterial hypertension (PH)-specific therapies are currently approved to treat SAPH^5^. Prior pilot studies and anecdotal experiences have yielded overall neutral results with pulmonary vasodilators in management of patients with SAPH^1,9–13^. However, comprehensive description of pulmonary hypertension, including hemodynamics, functional and echocardiographic findings, in SAPH is limited, especially description of real-world experiences and in context of ongoing treatment for sarcoidosis^14,15^. We aimed to characterize a large cohort of patients with newly diagnosed SAPH, factors that led to treatment with PH-specific therapy, and phenotypes associated with adverse clinical outcomes in follow-up. Because of the questionable validity of 6-minute walk distance (6MWD) as a surrogate marker for PH severity in the setting of pulmonary sarcoidosis^16^, we also included natriuretic peptide endpoints in our study.

## Methods

We identified patients using the Decision Support Repository, a Duke University Health System electronic data warehouse that aggregates clinical data of patients. Patient data available in the database include laboratory data, demographics, International Classification of Diseases-9 codes, medications, and computerized physician order entry logs; details of its design have been previously described^17^, We also reviewed electronic health records to collect sarcoidosis stage, pulmonary function test findings, vital signs, echocardiography and right heart catheterization findings. Our study population of interest consisted of patients evaluated at a single tertiary care referral center for pulmonary vascular disease between 1990–2010. Patients had biopsy-confirmed diagnosis of pulmonary sarcoidosis and a diagnosis of SAPH, defined as mPAP ≥ 25 mmHg at rest as measured by right heart catheterization. To exclude left heart dysfunction as a cause of pulmonary hypertension, patients with pulmonary capillary wedge pressure (PCWP) > 15 mmHg were excluded. Patients with missing values at baseline for 6MWD and N-terminal pro-brain natriuretic peptide (NT-proBNP) were excluded from statistical testing. The median follow-up period was 3 years.

We classified the initial PH management for each patient as one of the following 5 strategies: 1) Inhaled Monotherapy (inhaled treprostinil or iloprost); 2) Oral Monotherapy (phosphodiesterase-5 [PDE-5] inhibitor, endothelin receptor antagonist [ERA] or soluble guanylate cyclase [sGC] stimulator); 3) Parenteral Monotherapy (intravenous treprostinil or epoprostenol); 4) Initial Combination Therapy (more than one therapy); 5) No Therapy. Patient medication use was defined as whether a patient used a medication during their entire follow-up period. Missing values for medications including PH-specific therapy, immunosuppressant use (methotrexate, mycophenolate, hydroxychloroquine, or other), steroids at baseline and follow-up were considered to denote that they were not prescribed. We assessed the following endpoints: time to hospitalization or death, change in 6MWD, and change in NT-proBNP. Both 6MWD and NT-proBNP were assessed before and after initiation of PH-specific therapy. Pre-6MWD and pre-NT-proBNP were defined as the closest values within 3 to 9 months before beginning PH-specific therapy. Post-6MWD and post-NT-proBNP were the closest 6MWD value within 3 to 9 months after the patient’s first therapy was initiated. Right heart dysfunction and enlargement by echocardiography were categorized by an expert echocardiography reader categorically as ‘none’, ‘mild’, ‘moderate’, or ‘severe’.

The primary outcomes of interest were change in 6MWD and NT-proBNP. Changes in 6MWD and NT-proBNP by therapy group were reported using the following methods. A Kolmogorov–Smirnov (KS) test for normality was performed for both 6MWD and NT-ProBNP. The KS-test for normality was not rejected for the change in 6MWD (P = 0.15) and a paired t-test was used to assess for statistical significance. The KS-test for normality was rejected for the differences between Pre/Post NT-proBNP (P = 0.01), and therefore a sign test was used to compare median values of the cohorts. The study protocol was approved by the Duke University Health System institutional review board. Given our ability to use de-identified records from the electronic health record, a waiver of informed consent for this retrospective study was obtained from the institutional review board.

## Results

We identified 95 patients (76% female; 86% African American) with histopathological diagnosis of pulmonary sarcoidosis and new diagnosis of SAPH between 1990–2010 (Table 1). The mean age at the time of SAPH diagnosis was 52 years, and 70% of the cohort had Stage IV/advanced sarcoidosis. Almost all patients (99%) were symptomatic at baseline, and 77% of the cohort reported functional class III/IV symptoms. The median NT-proBNP at the time of initial evaluation was 910 pg/mL (Q1-Q3: 225–2807; reference < 225 pg/mL). Overall, 56 patients (59%) had either moderate or severe right ventricular (RV) enlargement and 55% had moderate or severe RV dysfunction. The median values for mPAP and PVR were 49 mmHg (Q1, Q3: 39, 60 mmHg) and 8.5 Wood units (Q1, Q3: 5.5, 12.7 Wood units), consistent with severe PH, and median cardiac index was also depressed (2.2 L/min/m^2^; Q1-Q3: 1.7–2.7 L/min/m^2^). The majority of patients was treated with medications for pulmonary sarcoidosis (61% on steroids and 18% on a non-steroidal immunosuppressant) on presentation to the PH clinic. Overall, 78% of patients in this cohort received PH-specific therapy for SAPH; 36 (37.9%) patients received initial oral monotherapy, 23 (24.2%) parenteral monotherapy, 4 (4.2%) inhaled monotherapy, 11 (11.6%) combined therapy, and 21 (22.1%) received no therapy.

**Table 1:** Baseline population characteristics of patients with SAPH grouped by PH-specific therapy initiation. Categorical variables expressed as N (%), and normally distributed continuous variables given as mean (SD), otherwise median (Q1, Q3).

	No PH therapy (N = 21)	Started on PH therapy (N = 74)	Total (N = 95)	P-value
Age at PH Diagnosis, years	49.4 ± 9.8	53.0 ± 11.2	52.2 ± 11.0	0.1
Women	12 (57.14%)	60 (81.08%)	72 (75.79%)	0.02
Race				1.0
Caucasian	2 (9.5%)	10 (13.5%)	12 (12.6%)	
African American	19 (90.5%)	63 (85.1%)	82 (86.3%)	
Other	0 (0%)	1 (1.4%)	1 (1.0%)	
BMI, kg/m^2^	28.4 (25.4, 36.1)	29.3 (24.8, 34.3)	28.6 (25.0, 34.3)	0.9
Smoker	8 (38.1%)	27 (36.5%)	35 (36.8%)	0.9
WHO Functional Class				0.1
Class 1	0 (0.00%)	1 (1.35%)	1 (1.06%)	
Class 2	6 (30.00%)	15 (20.27%)	21 (22.34%)	
Class 3	4 (20.00%)	34 (45.95%)	38 (40.43%)	
Class 4	10 (50.00%)	24 (32.43%)	34 (36.17%)	
Sarcoidosis Stage				0.2
0	2 (10.53%)	3 (4.05%)	5 (5.38%)	
1	2 (10.53%)	4 (5.41%)	6 (6.45%)	
2	4 (21.05%)	8 (10.81%)	12 (12.90%)	
3	1 (5.26%)	4 (5.41%)	5 (5.38%)	
4	10 (52.6%)	55 (74.3%)	65 (69.9%)	
DLCO, %	44.0 (24.0, 57.0)	32.0 (26.0, 45.0)	33.0 (26.0, 47.0)	0.2
Mean FVC, %	53.4 ± 20.2	56.5 ± 18.4	55.8 ± 18.7	0.6
**Immunosuppressants**				
Steroids	11 (52.4%)	47 (63.5%)	58 (61.0%)	0.4
Methotrexate	0 (0.0%)	6 (8.1%)	6 (6.3%)	0.3
Mycophenolate	0 (0.0%)	2 (2.7%)	2 (2.1%)	1.0
Hydroxychloroquine	2 (9.5%)	7 (9.5%)	9 (9.5%)	1.0
Systolic BP, mmHg	125.5 ± 22.3	126.7 ± 20.1	126.4 ± 20.5	0.8
**Biomarkers**				
NT-proBNP, pg/mL (N = 37)	510.5 (169.0, 5062.0)	1028.5 (254.0, 2311.0)	909.5 (225.0, 2807.0)	0.9
Echocardiography (N = 95)				
RV size				0.9
Normal	3 (14.3%)	16 (21.6%)	19 (20.0%)	
Mildly enlarged	5 (23.8%)	15 (20.3%)	20 (21.1%)	
Moderately enlarged	4 (19.1%)	16 (21.6%)	20 (21.1%)	
Severely enlarged	9 (42.9%)	27 (36.5%)	36 (37.9%)	
RV function				0.3
Normal	10 (47.6%)	21 (28.4%)	31 (32.6%)	
Mild dysfunction	1 (4.8%)	11 (14.9%)	12 (12.6%)	
Moderate dysfunction	5 (23.8%)	25 (33.8%)	30 (31.6%)	
Severe dysfunction	5 (23.8%)	17 (23.0%)	22 (23.2%)	
TAPSE, cm	1.9 ± 0.7	1.9 ± 0.8	1.9 ± 0.7	0.9
Pericardial effusion				0.2
None/trace	18 (85.7%)	69 (93.2%)	87 (91.6%)	
Mild	1 (4.8%)	4 (5.4%)	5 (5.3%)	
Moderate	2 (9.5%)	1 (1.4%)	3 (3.2%)	
Hemodynamics (N = 75)				
RA pressure, mmHg	10.0 (8.0, 15.0)	8.0 (5.0, 13.0)	8.0 (5.0, 14.0)	0.3
Mean PA pressure, mmHg	47.6±15.3	49.1 ± 12.0	48.8 (12.5)	0.7
Cardiac index, L/min/m^2^	2.2 (1.9, 2.8)	2.3 (1.7, 2.7)	2.2 (1.7, 2.7)	0.8
PVR, Wood units	6.1 (4.1, 12.3)	8.6 (6.3, 12.7)	8.5 (5.5, 12.7)	0.1

Abbreviations: PH (pulmonary arterial hypertension), BMI (body mass index), WHO (World Health Organization), DLCO (diffusing capacity of the lung for carbon monoxide), FVC (forced vital capacity), NT-proBNP (N-terminal pro-brain natriuretic peptide), RV (right ventricular), TAPSE (tricuspid annular plane systolic excursion), RA (right atrial), PA (pulmonary artery), PVR (pulmonary vascular resistance).

We then compared characteristics of SAPH patients based on initiation of PH-specific therapy (Table 1). Patients receiving therapy were 81% women as opposed to 57% in the no therapy group (P = 0.02) and had a higher PVR although not statistically significant (8.6 vs 6.1 Wood units, P = 0.10). There was no association between RV dysfunction and therapy, sarcoidosis stage, and mPAP (P > 0.2 for all). Subjects receiving parenteral monotherapy had the highest prevalence of moderate/severe RV dysfunction at baseline (19/23 or 83%), and those receiving no therapy had the lowest moderate/severe RV dysfunction (10/21 or 48%).

### Clinical Outcomes

The median time to hospitalization or death was 6 months (Q1-Q3: 3.0–12.0 months). Of the 64 patients (67%) who had a clinical event, stage IV sarcoidosis was present in 73% compared to 63% of those who did not experience hospitalization nor mortality (P = 0.69) (Table 2). Neither immunosuppressant, steroid, nor PH therapies were associated with having a clinical event. Baseline echocardiographic and hemodynamic characterization of RV function and PH was not associated with outcome, but follow-up NT-proBNP value was higher in those who died or were hospitalized (1258.0 vs. 262.0 pg/mL, P = 0.007).

**Table 2:** Baseline and follow-up characteristics for patients grouped by clinical event status at the end of study duration. Categorical variables expressed as N (%), and normally distributed continuous variables given as mean (SD), otherwise median (Q1-Q3).

	Death or hospitalization (N = 64)	No clinical event (N = 31)	Total	P-value
**Baseline**				
Age at PH diagnosis, years	50.9 (11.3)	55.1 (9.9)	52.2 (11.0)	0.08
Women	51 (79.7%)	21 (67.7)	72 (75.8%)	0.2
Race				1.0
Caucasian	8 (12.5%)	4 (12.9%)	12 (12.6%)	
African American	55 (85.9%)	27 (87.1%)	82 (86.3%)	
Other	1 (1.6%)	0 (0%)	1 (1.1%)	
BMI, kg/m^2^	28.5 (25.7, 36.1)	28.9 (21.3, 33.8)	28.6 (25.0, 34.3)	0.4
WHO functional class				0.6
Class 1	1 (1.6%)	0 (0%)	1 (1.1%)	
Class 2	12 (18.8%)	9 (30.0%)	21 (22.3%)	
Class 3	26 (40.6%)	12 (40.0%)	38 (40.4%)	
Class 4	25 (39.1%)	9 (30.0%)	34 (36.2%)	
Sarcoidosis stage				0.7
0	3 (4.8%)	2 (6.7%)	5 (5.4%)	
1	4 (6.4%)	2 (6.7%)	6 (6.5%)	
2	8 (12.7%)	4 (13.3%)	12 (12.9%)	
3	2 (3.2%)	3 (10.0%)	5 (5.4%)	
4	46 (73.2%)	19 (63.3%)	65 (69.9%)	
DLCO, %	33.0 (25.0, 47.0)	32.0 (27.0, 45.0)	33.0 (26.0, 47.0)	0.7
**Immunosuppresants**				
Steroids	39 (60.9%)	19 (61.3%)	58 (61.1%)	1.0
Methotrexate	5 (7.8%)	1 (3.2%)	6 (6.3%)	0.7
Mycophenelate	2 (3.1%	0 (0%)	2 (2.1%)	1.0
Hydroxychloroquine	5 (7.8%)	4 (12.9%)	9 (9.5%)	0.5
**Parameters at follow-up**				
NT-proBNP, pg/mL	1258.0 (365.0, 3286.0)	262.0 (150.5, 1810.0)	785.5 (232.0, 2828.5)	0.007
RV size				0.2
Normal	41 (64.1%)	26 (83.9%)	67 (70.5%)	
Mildly enlarged	6 (9.4%)	2 (6.5%)	8 (8.4%)	
Moderately enlarged	10 (15.6%)	1 (3.2%)	11 (11.6%)	
Severely enlarged	7 (10.9%)	2 (6.5%)	9 (9.5%)	
RV function				0.4
Normal	46 (71.9%)	25 (80.7%)	71 (74.7%)	
Mild dysfunction	3 (4.7%)	3 (9.7%)	6 (6.3%)	
Moderate dysfunction	11 (17.2%)	2 (6.5%)	13 (13.7%)	
Severe dysfunction	4 (6.3%)	1 (3.2%)	5 (5.3%)	
TAPSE, cm	1.7 (0.5)	1.8 (0.6)	1.8 (0.5)	0.7
RA pressure, mmHg	10 (5, 14)	7 (4, 13)	9 (4, 14)	0.3
Mean PA pressure, mmHg	48 (13)	45 (12)	47 (13)	0.4
Cardiac index, L/min/m^2^	2.4 (1.9, 3.0)	2.5 (2.2, 3.0)	2.5 (2.0, 3.0)	0.4
PVR, Wood units	8.4 (5.3, 11.5)	7.4 (4.1, 8.9)	8.1 (4.9, 10.5)	0.09

Abbreviations: PH (pulmonary arterial hypertension), BMI (body mass index), WHO (World Health Organization), DLCO (diffusing capacity of the lung for carbon monoxide), NT-proBNP (N-terminal pro-brain natriuretic peptide), RV (right ventricular), TAPSE (tricuspid annular plane systolic excursion), RA (right atrial), PA (pulmonary artery), PVR (pulmonary vascular resistance).

### Pre- and post-therapy initiation

**Figure 1:**
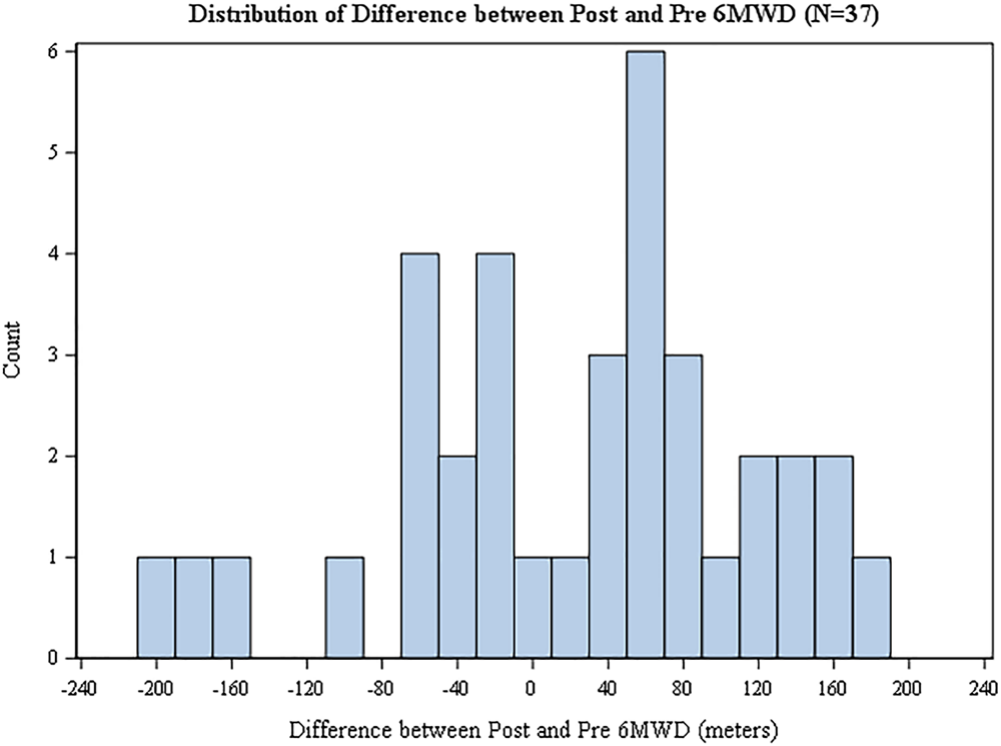
Distribution of change in 6MWD from pre- to post-initiation of PH-specific therapy in SAPH cohort. Positive values correspond to an increase in 6MWD. For display purposes, we exclude one outlier that falls outside of this range. Sensitivity analysis show that the results do not change based on this outlier.

**Figure 2:**
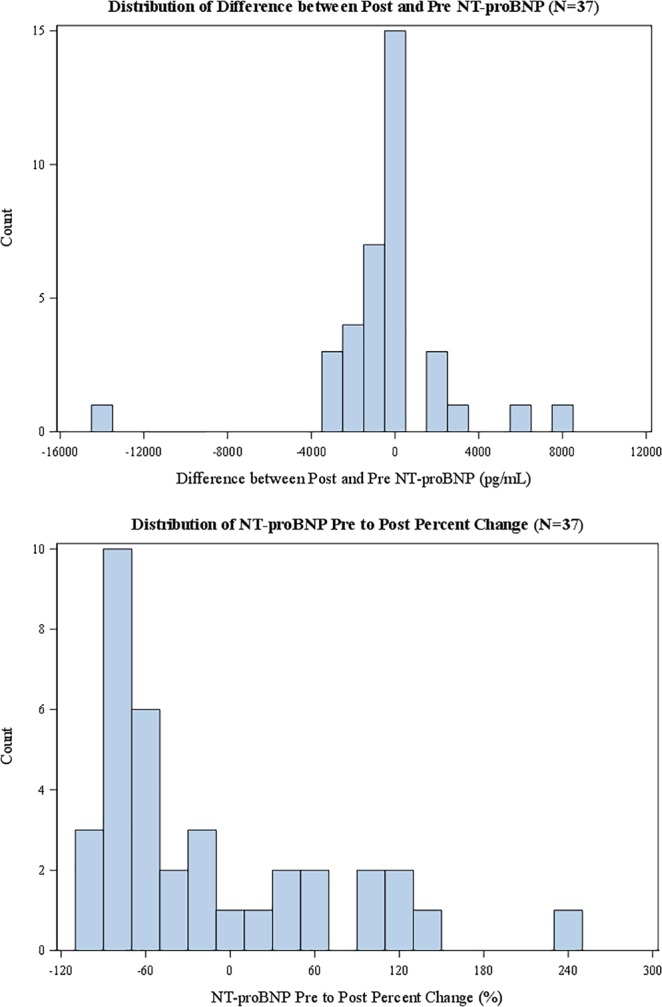
Distribution of change in NT-proBNP (top) and percent change in NT-proBNP (bottom) with PH-specific therapy in SAPH cohort. Positive values correspond to an increase in NT-proBNP. For display purposes, we exclude one outlier that falls outside of this range. Sensitivity analysis show that the results do not change based on this outlier.

Among the 74 patients receiving PH-specific therapy, 37 (50%) had non-missing 6MWD and NT-proBNP values in the pre-specified 3–9 months pre- and 3–9 months post-therapy initiation windows. Of this cohort, 33 patients continued on their original therapy throughout study follow-up. 6MWD did not significantly change over time (mean change = 10.8 ± 118.0 m, P = 0.29) (Fig. 1**)**, but NT-proBNP decreased after therapy was initiated (median change  = −387 pg/mL, P = 0.049) (Fig. 2, top) with a median percentage change of 51.2% once therapy was started (P = 0.049) (Fig. 2, bottom).

## Discussion

In this study, we characterized 95 patients with SAPH followed at a single academic center, the management of their PH, and their clinical outcomes. Unlike prior studies, we have included and described separately patients receiving and not receiving PH therapy to provide a more complete description of clinical course. We reported three major findings. First, this cohort had a high burden of morbidity. Almost 4 out of 5 patients with SAPH at the time of their initial diagnosis had significant functional limitations, reporting class III or IV symptoms, and the median time to hospitalization or death was only 6 months (not associated with pulmonary sarcoidosis stage), reflecting the disease burden associated with both pulmonary hypertension and stage IV pulmonary sarcoidosis^18^. The presence of stage IV sarcoidosis at time of SAPH diagnosis was not associated with whether a patient experienced hospitalization/mortality at follow-up. Among the 37 patients receiving PH-specific therapy, we observed a 51% improvement in NT-proBNP. Further, given the association between an elevated follow-up NT-proBNP and hospitalization/ death, it is possible that the elevated NT-proBNP may reflect persistent right heart dysfunction, the central determinant of survival in PH. This is consistent with a recent study by Boucly *et al*. in which hemodynamics improved with PH therapy in SAPH patients^14^. Like Boucly *et al*., we also found no change in 6MWD with PH-specific treatment in our SAPH population.

Several prospective pilot studies have been performed for various PH-specific therapies in SAPH with overall neutral/mixed results and have been summarized recently^5^. However, important limitations to these studies should be recognized to provide context. For example, in an open-label trial of ambrisentan, a high dropout rate (>50%) was observed for the 21 subjects, and a lower dose or placebo control arm would have provided further insight into the poor tolerability. In a double-blind, placebo-controlled trial, bosentan was well-tolerated and serial right heart catheterization demonstrated improvement in mean PA pressure and PVR, but 6MWD actually worsened in the bosentan group^11^. Retrospective studies of epoprostenol^19^, iloprost^12^, and sildenafil^20^ similarly have reported improvement in hemodynamics but no consistent direction of 6MWD change^13,21^. These mixed findings may be related to small sample sizes, the multifactorial etiologies of pulmonary hypertension in sarcoidosis patients, and co-existing disease processes, underscoring the need for careful phenotyping of patients and goals/endpoint selection to measure the contribution of PH to the patient’s overall condition. These studies coupled with our findings suggest that 6MWD may not be an appropriate measure of treatment success in SAPH.

Two studies reporting on characteristics of SAPH patients have been previously reported, the University of Chicago cohort (N = 26)^1^ and the United Kingdom cohort (N = 24)^10^. Both studies found high rates of mortality. Prostacyclin therapy was associated with no increased mortality and improved NT-proBNP in the University of Chicago patients, and pulmonary vasodilators in general were associated with improved hemodynamics and 6MWD in the United Kingdom patients. Right heart failure and significant lung fibrosis were associated with worse outcomes in this group. Our study adds a more complete description of clinical characteristics. We found that our SAPH patients had severe PH on presentation. The diagnosis of pulmonary hypertension in sarcoidosis patients can be challenging given the overlap of symptoms, and new-onset right heart failure may be the event that initiates workup in some patients^8^. Once diagnosed with SAPH, many factors are considered when assessing candidacy for treatment with PH-specific therapy including functional capacity, laboratory, imaging, invasive testing, and evaluation for concomitant left heart disease^22^. This study included providers specializing PH, whereas many patients with SAPH are managed by clinicians who do not routinely manage PH, and likely affects treatment decisions. In our cohort, gender was the only characteristic among demographics, disease-specific variables, and echocardiographic/hemodynamic descriptors of pulmonary hypertension that was associated with whether or not PH therapy was initiated (due to clinical impression of treatable PH). Although the reason for this is unclear, it is possible that women may be more likely to develop pulmonary hypertension due to pulmonary vascular disease as opposed to other etiologies of PH in the setting of pulmonary sarcoidosis. It is also possible that additional information including functional limitations, vasoreactivity^9,19^, and more refined imaging characteristics may be needed to prospectively identify the SAPH patients most likely to benefit from treatment.

Our study also assessed all-cause hospitalizations and death as clinical outcomes of interest. Patients who were started on intravenous, inhaled, or oral combination therapy for SAPH were much more likely to experience a clinical event in follow-up. Given prior evidence that these PH-specific therapies are overall well-tolerated and are associated with reduced NT-proBNP in our cohort, these findings are less likely related to adverse drug effect, and instead reflects a sicker subgroup of SAPH patients. Importantly, maintaining a low NT-proBNP at follow-up was associated with freedom from hospitalization/death and may represent an intermediate endpoint for patients with SAPH.

Our study has several important limitations related to the retrospective design. Because data was not collected prospectively, there was missingness of data that limited follow-up assessment of intermediate endpoint changes including 6MWD and NT-proBNP. Therapeutic decision-making was done by experts on an individual patient basis, and therefore difficult to generalize or study systematically in a retrospective fashion. Because parenteral therapy was the main therapeutic option in earlier years of the study, it is likely that this was overrepresented in our patient population as treatment choice. Further, to best understand treatment patterns of SAPH, an analysis of variation in practice by provider type (i.e., PH specialist) is necessary. Evidence to support clinical decisions in SAPH remains limited and optimal treatment is still unknown^6,8^.

## Conclusions

At a large academic center, patients with newly diagnosed SAPH have pulmonary sarcoidosis that spans all stages, and present with significant functional limitations and severe pulmonary hypertension. Although overall risks/benefits need further exploration, PH therapy initiation was associated with decreased NT-proBNP. Unadjusted analysis of follow-up NT-proBNP, but not 6MWD, was associated with death and hospitalization in SAPH patients and may be an important intermediate endpoint in the SAPH population.

## Data Availability

The datasets generated during and/or analysed during the current study are available from the corresponding author on reasonable request.
